# Gene regulation mediated by microRNAs in response to green tea polyphenol EGCG in mouse lung cancer

**DOI:** 10.1186/1471-2164-15-S11-S3

**Published:** 2014-12-16

**Authors:** Hong Zhou, Jayson X Chen, Chung S Yang, Mary Qu Yang, Youping Deng, Hong Wang

**Affiliations:** 1 Department of Mathematics, University of Saint Joseph, 1678 Asylum Avenue, West Hartford, CT 06117, USA; 2 Susan L. Cullman Laboratory for Cancer Research, Department of Chemical Biology and Centre for Cancer Prevention Research, Ernest Mario School of Pharmacy, Rutgers, The State University of New Jersey, 164 Frelinghuysen Road, Piscataway, NJ 08854, USA; 3 MidSouth Bioinformatics Center, Department of Information Science, George W. Donaghey College of Engineering and Information Technology, University of Arkansas at Little Rock, 2801 S. University Avenue, Little Rock, Arkansas, 72204, USA; 4 Joint Bioinformatics Graduate Program, University of Arkansas at Little Rock and University of Arkansas for Medical Sciences, Little Rock, Arkansas 72204, USA; 5 Rush University Cancer Center, and Departments of Internal Medicine and Biochemistry, Rush University Medical Center, Chicago, Illinois 60612, USA

## Abstract

**Background:**

Epigallocatechin-3-gallate (EGCG) has been demonstrated to inhibit cancer in experimental studies through its antioxidant activity and modulations on cellular functions by binding specific proteins. We demonstrated previously that EGCG upregulates the expression of microRNA (i.e. miR-210) by binding HIF-1α, resulting in reduced cell proliferation and anchorage-independent growth. However, the binding affinities of EGCG to HIF-1α and many other targets are higher than the EGCG plasma peak level in experimental animals administered with high dose of EGCG, raising a concern whether the microRNA regulation by HIF-1α is involved in the anti-cancer activity of EGCG *in vivo*.

**Results:**

We employed functional genomic approaches to elucidate the role of microRNA in the EGCG inhibition of tobacco carcinogen-induced lung tumors in A/J mice. By analysing the microRNA profiles, we found modest changes in the expression levels of 21 microRNAs. By correlating these 21 microRNAs with the mRNA expression profiles using the computation methods, we identified 26 potential targeted genes of the 21 microRNAs. Further exploration using pathway analysis revealed that the most impacted pathways of EGCG treatment are the regulatory networks associated to AKT, NF-κB, MAP kinases, and cell cycle, and the identified miRNA targets are involved in the networks of AKT, MAP kinases and cell cycle regulation

**Conclusions:**

These results demonstrate that the miRNA-mediated regulation is actively involved in the major aspects of the anti-cancer activity of EGCG *in vivo*.

## Background

The consumption of green tea, a beverage derived from the dried leaves of the *Camellia sinensis *plant, has a long history in Asian countries and is becoming more popular in Western nations. Accumulated data has suggested that the consumption of green tea is beneficial to human health, and some of the benefits are supported by results from the experimental studies. In particular, the cancer preventive activity of green tea has been extensively investigated and demonstrated in many organ sites in different animal models and cell line systems [1,2]. The most abundant and active anti-cancer constituent in green tea is (-)-epigallocatechin-3-gallate (EGCG). EGCG and other tea catechins are also referred to as green tea polyphenols for their carrying multiple phenolic groups with the ability to trap reactive oxygen species (ROS). Substantial studies have been conducted to uncover the cancer preventive mechanism of EGCG at the cellular and molecular levels. The results from these studies suggest that the treatment with EGCG or EGCG-rich tea extract leads to a wide range of responses, and the cancer prevention activity is likely to be mediated through multiple mechanisms that are a result of EGCG's direct scavenging of ROS and/or its physical interactions with specific proteins to modulate gene expression and cellular signaling. The effects of EGCG include the increment in detoxification capacity to prevent carcinogen-induced cellular damages [3-5], alteration in epigenetic modifications such as reducing the DNA hypermethylation-induced silencing of tumor suppressor genes [6-11], inhibition on tumor cell growth by inducing cell cycle arrest and apoptosis [2,12], anti-inflammation [4,13,14], and inhibition on tumor-associated angiogenesis [2,12,15].

However, most experimental evidences supporting anti-cancer mechanisms of green tea polyphenols including the ones mentioned above are mainly obtained from *in vitro *studies, and whether these mechanisms play significant roles in the cancer prevention/inhibition *in vivo *are largely unknown. To clarify this issue, studies using experimental animals are necessary. One well-characterized animal model used is the tobacco carcinogen-induced lung carcinogenesis in A/J mice. It has been shown by using this animal model that tumor multiplicity and size are effectively inhibited when mice are fed on a diet containing EGCG [2,16]. A/J mice treated with tobacco carcinogen such as 4-(methylnitrosamino)-1-(3-pyridyl)-1-butanone (NNK) or benzo[*a*]pyrene (B[a]P) develop lung adenoma within 20 weeks, and these tumors begin to progress to adenocarcinoma after 20 weeks [17-19]. When 0.5% green tea polyphenol extract was given to the NNK-treated A/J mice as drinking fluid for 32 weeks, the progression of adenoma to adenocarcinoma was inhibited [19]. This result was consistent with EGCG inhibiting the growth of the xenograft tumors of human lung cancer cell lines H1299 and H460 in nude mice [20]. In these *in vivo *studies, apoptosis was induced and pro-proliferation signaling (i.e. c-Jun and phospho-ERK1/2) were reduced in tumors, but not in normal lung tissues, after EGCG treatment [19,20]. Differential gene expressions have been profiled in the tumors from NNK- and B[a]P-treated mice fed on diet with or without green tea polyphenol extract for 20 weeks. Cell cycle regulation and inflammation were found to be the most impacted pathways by EGCG [21], suggesting that the anti-cancer activity of green tea polyphenols is mediated by inhibiting cell proliferation and anti-inflammation. These inhibitions are likely to be combinatory effects resulted from changes in the expressions of multiple genes induced by the green tea polyphenols [21].

An effective mechanism to regulate multiple classes of genes is through small regulatory RNAs called microRNAs (miRNAs) [22,23]. MiRNA exerts very fine-tune regulation on the expression of most protein-coding genes to optimize their expressions [22,23]. Upon binding to its target mRNA, miRNA can activate the Argonaute-catalyzed targeted cleavage of mRNA [24]; thus, miRNA predominantly acts to reduce the targeted mRNA [25]. Because miRNA recognizes the short segment usually in the 3' untranslated region of mRNA with imperfect complementarity, one miRNA is able to recognize a set of short RNA motifs featured with a common 5'-end sequence and variable 3'-end sequence, which increases the magnitude of the numbers of genes/mRNAs targeted by a miRNA [22,26]. Cellular miRNA pool forms a hierarchical regulatory network that is associated with cell identity and function, and the miRNA expression profile can be reset when the cell identity changes during development, differentiation, environmental challenge, and tumorigenesis [27]. In lung development, miRNA has been demonstrated to play critical roles. Different groups of miRNAs are expressed in developing and mature lung tissues, suggesting the distinct roles of these miRNAs in regulating cell growth and differentiation of lung tissue as well as maintaining normal lung functions [28]. For example, the miR-17-92 cluster promotes the proliferation of lung progenitors [28], whereas miR-34c, miR145, and miR-142-5p suppress lung cell growth [29]. In lung tumorigenesis, there are also evidences supporting the critical roles of miRNA. For example, miR-34c, miR145, and miR-142-5p, which suppress lung cell growth, are found to be repressed in both human and mouse lung cancer [29]. Furthermore, the overall miRNA levels in the oncogenic *K-ras*-induced mouse lung cancer are found to be reduced [30], which is consistent with the finding that lung-specific knockout of Dicer results in the abnormality of lung development and function [31]. Since the 22 base-active miRNA is initially expressed in a larger precursor fragment called pre-miRNA and is generated through the maturation process mediated by Dicer [32], there is no mature functional miRNA without Dicer. These data suggest that Dicer is a haploinsufficient tumor suppressor gene in lung, and the optimized level of miRNA is necessary to maintain the normal lung cell functions and phenotype [33].

Since we and others have demonstrated that EGCG can prevent lung carcinogenesis and inhibit lung cancer growth [2], we explored whether the cellular changes in lung cancer cells treated with EGCG are associated with alteration in miRNA expression. By studying the miRNA profiles in mouse and human lung cancer cells treated with EGCG, we found that the upregulation of miR-210 is the predominant miRNA event in response to the EGCG treatment, resulting in the reduced proliferation and anchorage-dependent growth [34]. This finding provides the evidence to support the importance of miRNA in the anti-cancer activity of EGCG. However, the upregulation of miR-210 by EGCG in the cultured lung cancer cell lines required higher concentration (i.e. over 20 μM) of EGCG than the highest plasma peak level (i.e. ~10 μM) detected in humans or animals administered with high dose of EGCG [2,35,36]. This raises a concern whether such a mechanism is effective *in vivo*, although a wide range of doses (i.e. 0.1-100 μM) are commonly used in the *in vitro *studies. In this study, in order to determine whether miRNAs such as miR-210 are involved in the cancer inhibition by EGCG *in vivo*, we employed the NNK-induced lung carcinogenesis in A/J mice fed on a diet with or without EGCG for miRNA and mRNA profile studies. Since the most effective inhibition induced by the EGCG treatment in this animal model is the inhibition of progression from adenoma to adenocarcinoma [19], one of the best windows to study the relevant EGCG-induced miRNA and mRNA profiles is on the adenoma cells before their progression to adenocarcinoma. Thus, the A/J mice were first treated with NNK to induce adenoma. After 19 weeks, the mice were fed a diet with or without EGCG for a short-term (i.e. one week) in order to study the miRNA and mRNA profiles before the progression. By using genomic and bioinformatics approaches to analyse the generated mRNA profiles, we uncovered that the most impacted pathways by the EGCG treatment are the networks related to AKT, NF-κB, MAP kinases, and cell cycle regulation. Through the miRNA profiles, we identified a group of miRNAs responsive to EGCG, and we further found that the potential targets of these miRNAs include genes in the regulatory networks of AKT, MAP kinases and cell cycle regulation. Such discoveries demonstrated that the regulation of miRNA is part of the anti-cancer activity of EGCG *in vivo*.

## Results

### Treatment with EGCG induces changes in miRNA expression in the NNK-induced mouse lung tumor

**Figure 1:**
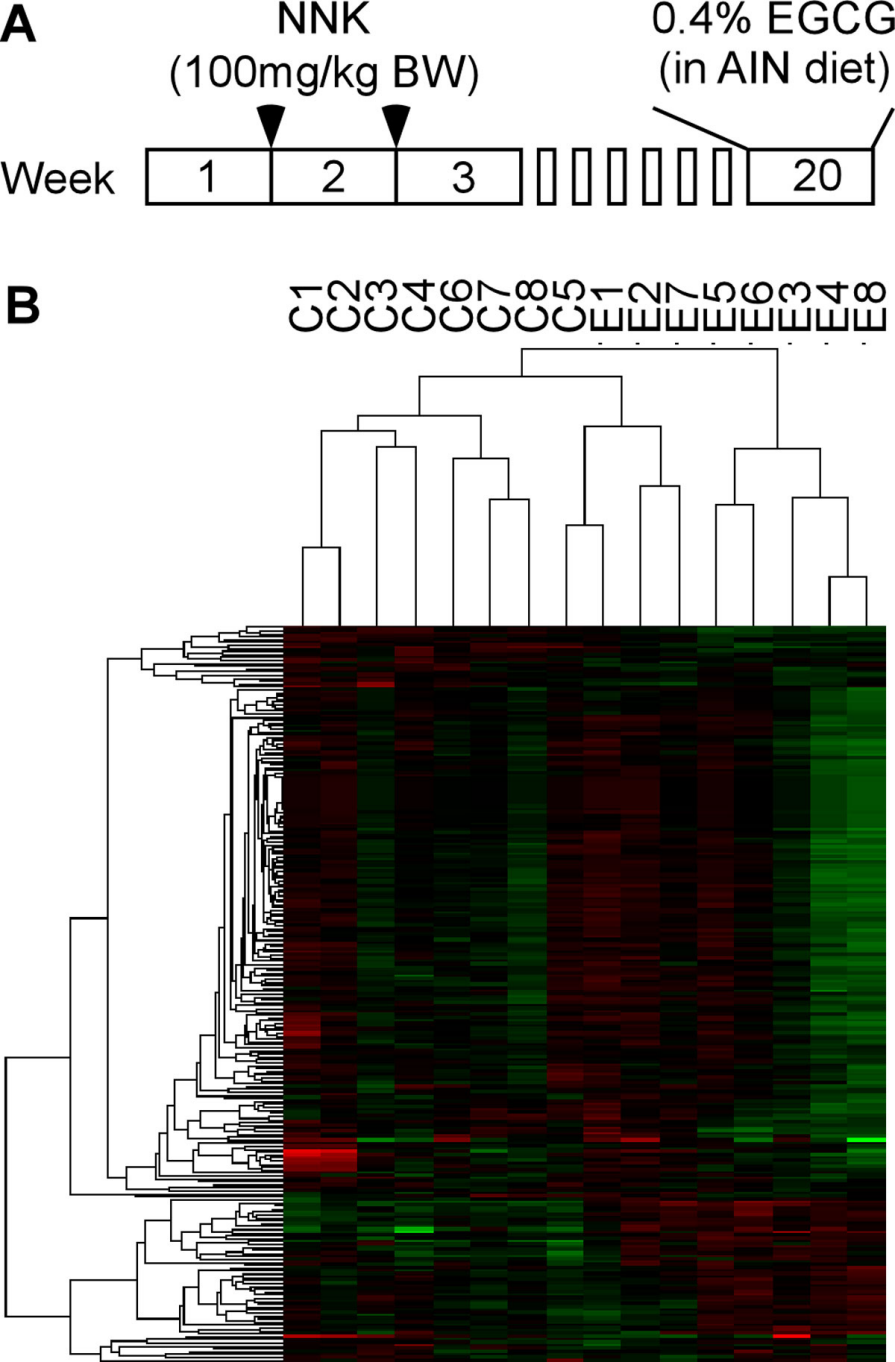
**A. Illustration of the animal experiment schedule**. B. Heat map of the cluster analysis of the miRNA profiles of the NNK-induced mouse lung tumors in the A/J mice fed on the control (C1-8) and EGCG diet (E1-E8).

We previously studied the miRNA expression profiles in response to the EGCG treatment using human and mouse lung cancer cell lines and found that the upregulation of miR-210 is the predominant miRNA event responding to the EGCG treatment and the overexpression of miR-210 inhibits lung cancer cell proliferation [34]. The upregulation of miR-210 by EGCG was demonstrated to be mediated through the stabilization of HIF-1α by EGCG. The Kd of the EGCG binding to HIF-1α is 3.47 μM [34], which is compatible to the highest plasma peak EGCG level (i.e. 7-10 μM) in the experimental animals administered with diet containing high dose of EGCG [2,35], suggesting the possibility that miR-210 may play a role inhibiting lung cancer *in vivo*. However, what concentration can be reached inside the cells and whether the duration is long enough to lead to physiological significance remains to be unclear. Herein, to address this possibility, we employed the NNK-induced lung carcinogenesis in A/J mice to study the miRNA profiles in mice fed on the control and EGCG diets in an effort to evaluate the role of miR-210 and other miRNAs *in vivo*. We treated 30 female A/J mice with NNK to induce lung adenoma according to previously established procedure [19]. At 19 weeks after the first NNK injection when the adenoma is expected to be induced at 100% incidence and readily to progress to adenocarcinoma, mice were randomly separated into the control and EGCG treatment groups (15 per group). While the control groups remained on AIN93M diet, the EGCG treatment group received 0.4% EGCG in diet for 1 week (Figure 1A). We expect that through this short-term treatment, the EGCG-treated adenomas would undergo some responses including changes in the expression of some genes that play a role in the EGCG-induced cancer inhibition. At the endpoint, we excised the tumors from lung under dissecting microscope, and obtained high quality and quantity RNAs from 8 control and 8 EGCG-treated samples. These samples were first used for the miRNA microarray to obtain the miRNA profiles. We conducted cluster analysis on the miRNA profiles and found that these 16 samples can be separated into three major clusters: all controls except C5 were in one cluster (control cluster), control C5 and 3 EGCG-treated samples (E1, E2, and E7) were in one cluster that is relatively close to the control cluster, and the rest 5 EGCG-treated samples were in the other cluster (responsive cluster) (Figure 1B). Since E1, E2 and E7 were between control and responsive groups, they represented a weak responsive group. Such a distribution actually presented the different responses of individual mice although we do not know what caused the difference.

By analysing the difference between the control and treatment groups (all 16 samples), we found 5 miRNAs were upregulated by at least 1.0 fold (P-value ≤ 0.01) and no miRNA was downregulated by at least 1.0 fold (P-value ≤ 0.01) in response to the EGCG treatment (Table 1). If we reduced the selection condition to 0.5 fold change, it was found that 12 miRNAs were upregulated in response to the EGCG treatment (the expression level is 1.5 fold or above (≥1.5) of the controls and P-value ≤ 0.01) (Table 1), and 9 miRNAs were downregulated (the expression level is 0.75 fold or lower (≤0.75) of the controls and P-value ≤ 0.01) (Table 2). When we excluded the 3 weak responsive samples from the treatment group and performed the same comparison between the 8 controls and the 5 responsive treatment samples, the result remained the same, suggesting that the identified EGCG-regulated miRNAs are the predominant events. To our surprise, miR-210, found upregulated by EGCG in *in vitro *experiment, ranks right behind the 12 upregulated miRNAs in this *in vivo *study (Table 1), but it was excluded because the large associated P-value indicates that its upregulation may not be statistically significant in all samples. By examining the raw data, miR-210 in the treatment group is upregulated about 1.44 fold and the associated P-values are 0.04 and 0.15 in the two comparisons using the 8 and 5 treatment samples respectively. This, however, might suggest that some animals did not intake sufficient EGCG to reach the level required for the activation of miR-210. Thus, the regulation of miR-210 by EGCG is unlikely to play a significant role *in vivo *in all animals at the dose used in this experiment. On the other hand, using miR-210 as the reference, we believe that the 12 upregulated miRNAs in Table 1 which ranked higher than miR-210 and are ubiquitously up in all samples with a P-value less than 0.01, are high confident miRNAs targeted by EGCG, and the 9 downregulated miRNAs in Table 2 that are identified by the same standards are also the targets with high confidence.

**Table 1:** The EGCG-upregulated miRNAs in the NNK-induced A/J mouse lung tumor.

miRNA name (Release 17)†	Accession Number	miRNA name (Release 20)†	Fold Changes	P-Value (8)‡	P-Value (5)ǁ	FDR (8)‡
mmu-miR-2137	MIMAT0011213	mmu-miR-2137	2.88	2.37E-07	1.24E-03	1.55E-04
mmu-miR-449a	MIMAT0001542	mmu-miR-449a-5p	2.47	2.21E-03	9.74E-03	5.79E-02
mmu-miR-144	MIMAT0000156	mmu-miR-144-3p	2.30	8.95E-04	3.06E-03	3.67E-02
mmu-miR-486#	MIMAT0003130	mmu-miR-486-5p	2.10	6.79E-04	1.89E-03	3.18E-02
mmu-miR-3107#	MIMAT0014943	mmu-miR-3107-5p	2.10	6.79E-04	1.89E-03	3.18E-02
mmu-miR-193	MIMAT0000223	mmu-miR-193a-3p	2.08	9.64E-03	2.21E-02	1.66E-01
mmu-miR-5130	MIMAT0020641	mmu-miR-5130	1.82	1.01E-04	6.02E-03	9.42E-03
mmu-miR-2861	MIMAT0013803	mmu-miR-2861	1.75	1.89E-05	1.45E-03	2.48E-03
mmu-miR-511-3p	MIMAT0017281	mmu-miR-511-3p	1.64	1.67E-02	6.86E-02	2.06E-01
mmu-miR-763	MIMAT0003896	mmu-miR-763	1.57	1.02E-03	9.37E-03	3.75E-02
mmu-miR-3473	MIMAT0015645	mmu-miR-3473a	1.56	4.12E-03	1.78E-02	9.00E-02
mmu-miR-211*	MIMAT0017059	mmu-miR-211-3p	1.53	1.19E-04	2.64E-03	9.73E-03
mmu-miR-210	MIMAT0000658	mmu-miR-210-3p	1.44	3.92E-02	1.59E-01	3.52E-01

† Based on http://www.mirbase.org

‡ Comparison includes all 8 controls and all 8 EGCG-treatment samples.

ǁComparison includes all 8 controls and 5 responsive EGCG-treatment samples.

# These 2 miRNAs, miR-486 and miR-3107, are recognized by the same probe.

**Table 2:** The EGCG-downregulated miRNAs in the NNK-induced A/J mouse lung tumor.

miRNA name (Release 17)†	Accession Number	miRNA name (Release 20)†	Fold Changes	P-Value (8)‡	P-Value (5)ǁ	FDR (8)‡
mmu-miR-696	MIMAT0003483	mmu-miR-696	0.54	2.66E-03	3.65E-02	6.33E-02
mmu-miR-449c	MIMAT0003460	mmu-miR-449c-5p	0.58	2.70E-06	9.81E-04	8.86E-04
mmu-miR-7a	MIMAT0000677	mmu-miR-7a-5p	0.60	1.67E-03	3.66E-03	5.12E-02
mmu-miR-205	MIMAT0000238	mmu-miR-205-5p	0.62	3.03E-01	3.86E-03	7.56E-01
mmu-miR-450a-2*	MIMAT0004789	mmu-miR-450a-2-3p	0.63	1.00E-04	7.93E-03	9.42E-03
mmu-miR-1199*	MIMAT0017333	mmu-miR-1199-3p	0.63	1.40E-04	1.10E-02	1.02E-02
mmu-miR-374c	MIMAT0014953	mmu-miR-374c-5p	0.63	8.79E-03	1.62E-02	1.56E-01
mmu-miR-218	MIMAT0000663	mmu-miR-218-5p	0.64	1.72E-03	3.82E-03	5.12E-02
mmu-let-7b*	MIMAT0004621	mmu-let-7b-3p	0.66	4.01E-04	4.74E-03	2.19E-02

† Based on http://www.mirbase.org

‡ Comparison includes all 8 controls and all 8 EGCG-treatment samples.

ǁComparison includes all 8 controls and 5 responsive EGCG-treatment samples.

### Treatment with EGCG induces changes in mRNA expression in the NNK-induced mouse lung tumor

**Figure 2:**
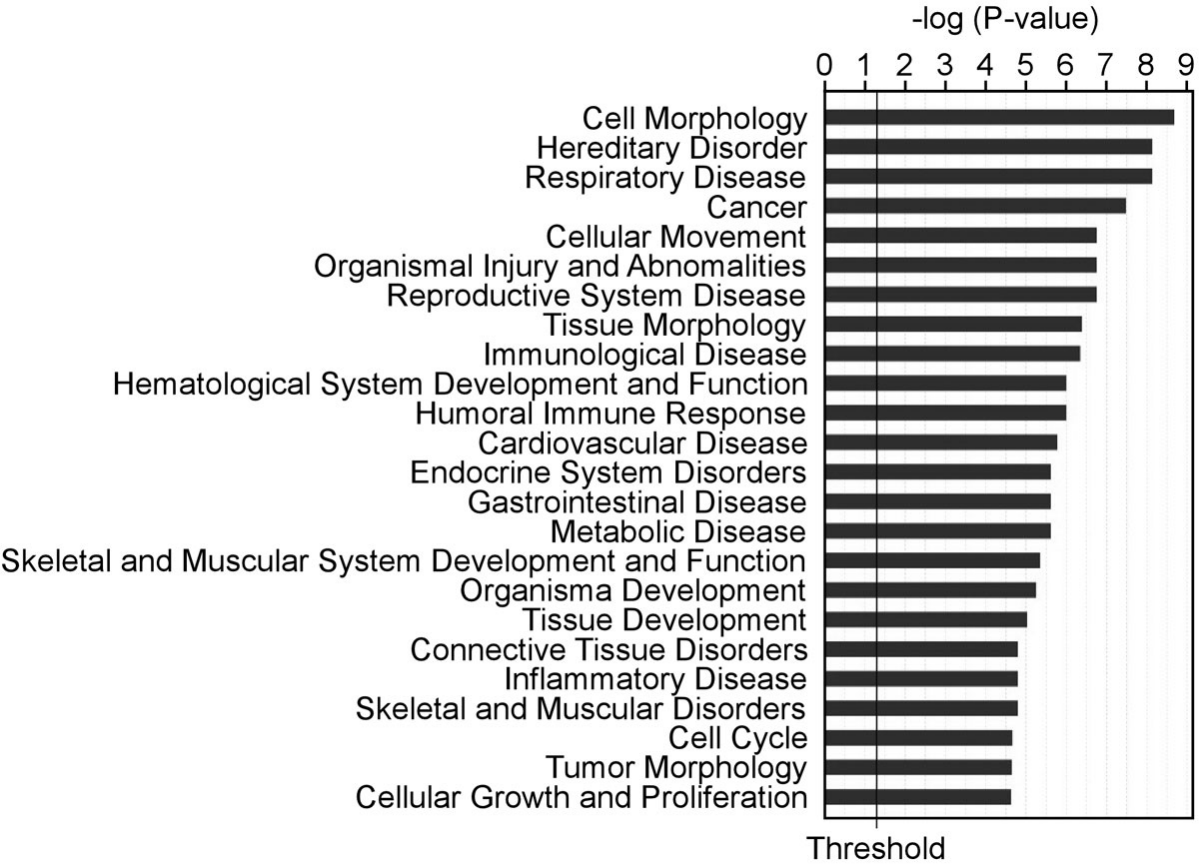
**Gene functional associations by Gene Ontology (GO) analysis of the changes in the mRNA expression profile in response to the EGCG treatment**.

To explore the roles of the EGCG-responsive miRNAs, we next aimed at uncovering the miRNA targeted genes. For this purpose, we selected RNA samples from 3 controls and 3 responsive samples for the mRNA microarray and obtained the mRNA expression profiles. By analysing the differentially expressed mRNA between the control and the EGCG-treated (with the condition of ≥1.0 fold change and P-value ≤ 0.01), we found that there were 295 and 71 mRNAs up- and down-regulated, respectively (Additional file 1 and Additional file 2), in response to the EGCG treatment. To understand the impact of these genes on the cellular functions, we analysed the functional associations of these 366 genes using Ingenuity Pathway Analysis. The result showed that the top associated functions of these genes include the regulation of morphology, movement, inflammation, development and proliferations (Figure 2), which are the relevant functions in tumor growth, progression, and metastasis. Further analysis revealed 18 pathways, and the top 4 relevant pathways are in the networks centralized by AKT, NF-κB, MAP kinases and cell cycle regulators (Additional files 3, 4, 5, 6), supporting the roles of EGCG in cell proliferation inhibition and anti-inflammation. Targeting these networks by EGCG are actually consistent with the literature supporting the roles of EGCG in inhibiting cell cycle and inflammatory response (reviewed in [2,37]). Indeed, a previous study using green tea polyphenol diet to treat A/J mice bearing the NNK-induced lung tumor for 20 weeks, a long term treatment, also revealed the modulation on the cell cycle: a group of 88 genes were identified as the responsive genes and a major associated network of these 88 genes was the cell cycle regulation [21]. Therefore, in both short- and long-term treatments, the inhibition on cell proliferation is the common mechanism.

### EGCG regulates cellular functions mediated through its regulations on miRNAs

We identified the expression changes of the 21 miRNAs in the NNK-induced lung tumor in response to the EGCG treatment in the above study. While we did not obtain evidence to support the role of miR-210 that is upregulated in the *in vitro *cultured lung cancer cells treated with EGCG, these 21 miRNAs represent the ones that are involved in the *in vivo *mechanisms of the EGCG inhibition of lung cancer. To explore the roles of these miRNAs in the EGCG-treated lung tumor, we used the computational approach to identify the potential targets of these miRNAs and correlated the predicated targets with the mRNA profiles based on the principal that miRNA downregulates its targeted mRNA. By using three highly confident and commonly used miRNA target prediction computation programs [38] as described in the Methods, we identified a group of 26 genes that are predicated to be the targets of the EGCG-regulated 21 miRNAs, and the changes of their expression levels are inversely correlated to the changes of miRNAs (Table 3). To evaluate the roles of these miRNA targeted genes, we performed Ingenuity Pathway Analysis for these 26 genes and revealed an interaction network that is centralized by IGFBP5 and is involved in the regulation of AKT and ERK1/2 (Additional file 7). This network includes some players in the networks of AKT, MAP kinase, and cell cycle regulation, which was identified as the top functional networks regulated by EGCG. Therefore, this result indicates that miRNA is involved in the major pathways of the anti-cancer activity of EGCG *in vivo*.

**Table 3:** Genes regulated by EGCG potentially mediated through miRNA.

mRNA coding Genes		Targeted by miRNA	
**Gene Name**	**Changes in expression level**	**miRNA name (Release 20)**	**Changes in expression level**

AACS	up	mmu-miR-696	Down

ACSL1	up	mmu-mir-205-5p mmu-miR-218-5p, mmu-miR-449c-5p	Down

AMMECR1	down	mmu-miR-144-3p	Up

ANKRD34B	down	mmu-miR-511-3p	Up

ART3	up	mmu-miR-374c-5p	Down

Ccdc22	down	mmu-miR-763	Up

Ccne2	down	mmu-miR-144-3p mmu-miR-34b-5p mmu-miR-449a-5p	Up

Cd19	up	mmu-miR-450a-2-3p	down

CENPF	down	mmu-miR-211-3p	Up

cnr1	up	mmu-miR-374c-5p	down

DCN	up	mmu-miR-374c-5p	down

EFNA5	up	mmu-miR-374c-5p	down

elmod1	up	mmu-miR-449c-5p	down

GAS1	up	mmu-miR-374c-5p mmu-miR-449c-5p	down

ELOVL6	up	mmu-miR-374c-5p	down

IGFBP5	up	mmu-miR-450a-2-3p	down

LASP1	up	mmu-miR-218-5p	down

NRN1	down	mmu-miR-34b-5p mmu-miR-449a-5p	Up

PER2	down	mmu-miR-211-3p	Up

per3	down	mmu-miR-3068-3p	Up

pfn2	up	mmu-miR-7a-5p	down

rbm24	up	mmu-miR-374c-5p	down

RBPMS2	up	mmu-mir-205-5p	down

SYNPO2	up	mmu-let-7b-3p	down

ZCCHC2	down	mmu-miR-144-3p	Up

ZFHX4	up	mmu-miR-449c-5p mmu-miR-450a-2-3p	down

## Discussion

In this study, we used the functional genomic approaches to explore the gene regulation mediated through miRNAs in response to the EGCG treatment in mouse lung tumor. We identified 21 miRNAs whose expressions are regulated by EGCG. By analysing the functional ontology of the potential targets of these 21 miRNAs, we found that the target genes include the key components in the signaling regulation pathways centralized with AKT, MAP kinase, and cell cycle regulators. Since these pathways play important roles in carcinogenesis, our results support the importance of miRNA in the cancer inhibition by EGCG *in vivo*.

By using genomic and bioinformatics approaches to analyse the mRNA profiles, we found that the EGCG-induced expression profile change *in vivo *constitutes regulatory networks which impact cellular signalings involving AKT, NF-κB, MAP kinases, and cell cycle regulation, consistent with the well-characterized inhibitions on proliferation and inflammation by EGCG. The inhibition on cell proliferation by EGCG has been demonstrated in different cancer cells and animal models [2,12,37]. Particularly, our data is consistent with our previous finding that the pro-proliferation signalling, such as the activation of MAP kinase (i.e. phospho-ERK1/2), was significantly reduced in tumor tissues but not in normal lung tissues after EGCG treatment [19,20]. The regulation on AKT signaling has been reported in many studies to be through various mechanisms including the direct downregulation of AKT mRNA and inhibition of AKT activation as well as the indirect inhibition of upstream signaling such as the activations of receptor tyrosine kinases, c-Met, EGFR and IGFR [2,12,37,39]. We did not find the expression level change in AKT, but our data rather suggested that the effect on AKT could be resulted from multiple mechanisms simultaneously. The regulation on NF-κB is also consistent with previous findings that EGCG displays anti-inflammation activity through inhibiting the inflammatory response master regulator NF-κB [12]. Like the regulation on AKT, the network reveals that NF-κB can be influenced by multiple pathways induced by EGCG: blocking the activation of NF-κB by quenching ROS [40], inhibiting the upstream regulators such as PI3K/Akt and MAP kinases [12], or inhibiting Pin1, a NF-κB chaperon protein [41]. However, the mRNA profile analysis did not find evidence for the involvement of pro-apoptosis and anti-angiogenesis, though both of which are demonstrated in other studies as the cancer inhibition mechanisms of EGCG [2,37]. Actually, the pro-apoptosis and anti-angiogenesis were not found in the mRNA profile analysis in the study using the long-term EGCG treatment either [21]. There are several possibilities that these two mechanisms were not identified by the expression profile analyses. First, the caspase-mediated initiation of apoptosis acts through the proteinase-mediated signaling cascade which can be independent of transcriptional activation or suppression. Second, the endothelial cells are accounted only for a small fraction in the tumors and the related changes are too weak to be recognized. Third, the dose of EGCG used in this study might not reach the effective level to cause these actions. Nevertheless, the miRNA and mRNA expression profile analysis provides trustworthy data to support that the *in vivo *mechanism of cancer inhibition of EGCG, and the identified targeted pathways are consistent with the data in the literature.

The major goal of this study was to explore the roles of miRNA in the anti-cancer activity of EGCG *in vivo*. We found that the EGCG-regulated miR-210 identified in our previous *in vitro study *[34] is upregulated to 1.44 fold with a large P-value, indicating the lower confidence on the upregulation of miR-210 in this study. Instead, we identified 21 miRNAs with high confidence. But the levels of these 21 miRNAs were not found to be regulated by EGCG in the cultured lung cancer cell lines, including CL13 mouse lung cancer cell line derived from the NNK-induced mouse lung cancer [18] in our previous study [34]. The discrepancy could be resulted from the differences in the levels of the oxidative stress and EGCG-binding proteins between primary tumor and the in vitro cultured cells. For example, the antioxidant activity of EGCG might play more important roles in the *in vitro *cultured cells that are usually exposed to higher levels of oxidative stress than in primary tissues [42]. Another possibility could be due to that the effective action of EGCG is on the adenoma progression to adenocarcinoma [19], and adenoma are the targeted cells in our current study whereas lung cancer cell lines are derived from advanced cancers (i.e. invasive adenocarcinomas). In addition, we cannot completely exclude the possibility that these changes take place in non-cancer cells. However, it seems unlikely because the majority of the cells in the isolated lung tumor tissues are dysplastic adenoma cells [18,19]. No matter what caused the difference between the *in vivo *and *in vitro *studies, our finding of the 21 EGCG-regulated miRNAs in this *in vivo *study strongly supports the importance of the use of experimental animals in the exploration of the anti-cancer mechanism of EGCG. Our result also supports that functional genetic and proteomic analysis is an effective approach to uncover the systematic responses in a complicated system such as primary tumor.

One long-standing unanswered question in the field is whether the cancer inhibition by EGCG *in vivo *is mediated by the mechanisms that are identified in the *in vitro *studies in which higher concentrations of EGCG (e.g. 50-100 μM) are commonly used. EGCG could target multiple pathways through different direct binding proteins [2]. In our previous study in which the miR-210 was identified as the major EGCG-regulated miRNA, we demonstrated the direct binding of EGCG to HIF-1α [34]. However, these direct binding proteins are targeted by EGCG at a wide range of binding affinities [35]; thus, the cellular functions targeted by EGCG are largely dependent on the doses used in the experiments. In most cases, to be effective *in vitro*, higher concentration of EGCG such as 20-100 μM are required. In contrast, the maximum plasma peak levels of EGCG in human and animal models administered with higher doses through drinking or diet is less than 10 μM [2,35]. The plasma concentration of EGCG is probably the most important factor in evaluating the working mechanism of EGCG *in vivo*. For example, the affinity for EGCG to bind HIF-1α is about 4.7 μM. To activate HIF-1α for upregulating miR-210 in the cultured lung cancer cells so as to produce significant effect, at least 20 μM EGCG in the culture medium is required [34], suggesting that a higher concentration of EGCG is necessary to reach the effective concentration inside the cells through free diffusion. Although we cannot exclude the cases that EGCG can function on the cell surface, be transported into cells by a transporter, or reach higher concentration in local tissues than the plasma level, a mechanism that requires higher dose should be further evaluated using the *in vivo *experiment. In this study, by analysing the profiles of miRNAs and mRNAs in the NNK-induced lung tumors in A/J mice fed on EGCG, we found that there was modest upregulation of miR-210 (an increase to 1.44 fold on average after the EGCG treatment). However, such upregulation of miR-210 manifests large differences among individual mice. Such differences might be due to that some mice failed to intake sufficient EGCG to reach the required level. Thus, this data suggests that miR-210 is unlikely to play a critical role in the EGCG-induced lung cancer inhibition *in vivo*.

The miRNA targeted genes were identified by the combination of computation method and the correlated expression changes in miRNAs and mRNAs. Indeed, the miRNA target identification traditionally relies on the computation method because the involved genomic information is too large to be easily handled via the experimental study [38]. Based on the validation using the genome-wide experimental studies, the computation predictions have been proven to be reliable with high confidence [43,44]. However, the rates of the false positive and negative calls remain high for each method. To overcome this pitfall, multiple predication methods were used to reduce the false calls. Since the next generation sequencing technology is well-developed and the related cost is reducing, the real target genes of a specific miRNA in a given context can be identified by sequencing the RNA products generated using the immunoprecipitated Argonaute protein-RNA complex [45]. Thus, a future study on the identification of the miRNA target genes using experimental approaches to validate our findings is under consideration.

## Conclusion

We demonstrated, for the first time, that EGCG induces miRNA profile changes in the NNK-induced A/J mouse lung tumor. The targeted genes of these miRNAs are involved in the regulatory network that plays an important role in the anti-cancer activity of EGCG *in vivo*. Furthermore, this *in vivo *study provides valuable data that is different from the result obtained from the *in vitro *studies, which further emphasizes the importance of animal models in the evaluation of cancer inhibitory mechanism of EGCG.

## Methods

### Animal experiment and treatment

Mouse lung carcinogenesis was induced by NNK in the susceptible A/J mice as described previously [19]. The experimental procedures were conducted in accordance to the animal protocol (Protocol No. 91-024) approved by the Animal Care and Facilities Committee of Rutgers, the State University of New Jersey. Thirty female A/J mice at the age of 4 weeks were purchased from the Jackson Laboratory (Bar Harbor). After arrival at the animal facility, mice were housed in the room at room temperature (20 ± 2°C) with a relative humidity of 50 ± 10% and with an alternating 12 h light/dark cycle throughout the duration of the study. One week later, mice diet was switched from lab chow to the purified AIN93M diet (Research Diet), the standard purified rodent diet with the defined nutrients by American Institute of Nutrition (AIN) committee. At the age of 6 weeks, mice were treated with a dose of NNK (100 mg/kg body weight, i.p.) (Chemsyn Science Laboratories) (Figure 1A). After a week, mice were treated with another dose of NNK (100 mg/kg body weight, i.p.). Mice were monitored daily during and after the treatments. At 19 weeks after the first NNK injection, mice were separated randomly into 2 groups (15 mice per group): one group remained on the AIN93M diet as the control, and the other group was fed the purified diet containing 0.4% EGCG (prepared by Research Diet) as the treatment. EGCG (94% purity) was a gift from Dr. Yukihiko Hara (Mitsui Morin Co.). After one week of the EGCG treatment, both groups of mice were sacrificed by CO_2 _asphyxiation and the lungs were removed. One leaf of lung from each mouse was inflated by PBS and fixed in 10% buffered formalin for pathology analysis. Visible tumors in the rest of lung tissue were carefully separated from the adjacent normal lung tissues under dissecting microscope and stored in RNAlater Solution (Ambion) at -80°C for RNA extraction. Because these tumors were very small (normally 0.1-0.3mm in diameter), we combined all tumors (an average of 20 per mouse) from one mouse as one sample.

### RNA purification and microarray analyses

Total RNA was purified from the tumor samples stored in RNAlater Solution using the miRNeasy kit (Qiagen) according to the manufacturer's protocol once the development of adenomas were validated by the histopathological analysis. Based on the quality and quantity of the total RNA samples, RNA samples from 8 mice fed on the controls diet and 8 on the 0.4% EGCG diet were used for the miRNA profiling. To obtain the miRNA expression profiles, miRNA samples were analyzed by the miRNA microarray conducted by Ocean Ridge Biosciences using Multispecies MicroRNA Array Chip covering miRBase Release 17.0. Data collection and analysis were also conducted by Ocean Ridge Biosciences using the standard method for microarray analysis. Briefly, for the data normalization, the normalization factor (N) for each microarray was obtained by the 20% trim mean of the species (human/mouse) probes intensities above threshold in all samples. The log2-transformed spot intensities for all probe features on the array were normalized by subtracting N from each spot intensity and scaled by adding the grand mean of N across all microarrays. P-value and false discovery rate (FDR) were calculated for all samples.

The mRNA expression profiles of 3 controls and 3 EGCG-treated samples were analysed by microarray using Affymetrix GeneChip Mouse Gene 1.0 ST Array by Functional Genomics Core at Cancer Institute of New Jersey. Data collection and analysis were also performed by the core according to Affymetrix array standard procedure.

### Bioinformatics analyses

The differential expressions of miRNA in tumors from mice on the control AIN93M diet or the 0.4% EGCG diet were analyzed by the Ocean Ridge Biosciences as described above. Briefly, the differentially expressed miRNAs were ranked by the smallest P-value and FDR value and then by the fold of change. The differential expressions of mRNA were analyzed using the BRB-Array Tool v4.4 beta 1 http://linus.nci.nih.gov/BRB-ArrayTools.html with the normalization by reference for single channel data. For the mRNA microarray data, the up- or down-regulated genes are defined to be those whose mRNA expression levels were increased or decreased in all three EGCG-treated samples compared to three controls and the average change must be at least 1.0 fold. P-value and FDR were calculated for all samples.

The potential target genes of a specific miRNA were identified using three common computation programs that are proved to predict the miRNA targets with better accuracy based on proteomic data [38]: miRDB (http://mirdb.org/miRDB/; [46,47]), Diana microT v3.0 (http://diana.cslab.ece.ntua.gr/microT/; [48,49]), and TargetScan 5.2 for mouse http://www.targetscan.org/mmu_50/. Only the gene which was identified as a candidate of a specific miRNA by at least two programs was classified as the candidate target gene. The candidate target genes were further identified as the miRNA target by the correlation of the expression level changes found in the miRNA and mRNA expression profiles; the up- or down-regulation in the mRNA expression level of a target gene should correspond to the opposite miRNA change.

To sort out the most impacted cellular functions, we loaded the overall differentially expressed genes and the identified miRNA targets to the Ingenuity Pathway Analysis http://www.ingenuity.com for further pathway analyses.

## Competing interests

The authors declare that they have no competing interests.

## Authors' contributions

All authors participated in the design, analysis of the data, interpretation of the results, and review of the paper. JXC, HW conducted the experiments. HZ, MQY and YD performed the bioinformatics analyses. HW, CSY and HZ drafted the paper. All authors read and approved the final version of the manuscript.

## Supplementary Material

Additional File 1 The EGCG-upregulated mRNAs in the NNK-induced A/J mouse lung tumor Click here for file

Additional File 2 The EGCG-downregulated mRNAs in the NNK-induced A/J mouse lung tumor Click here for file

Additional File 3 Top impacted pathway centralized with the regulation of AKT by the EGCG-induced genes in the NNK-induced mouse lung tumors. Shadowed shapes represent the EGCG targeted genes. Dashed lines represent the indirect interactions. The arrows represent the interaction directions. Click here for file

Additional File 4 Top impacted pathway centralized with the regulation of NF-κB and cytokines/chemokines by the EGCG-induced genes in the NNK-induced mouse lung tumors. Shadowed shapes represent the EGCG targeted genes. Dashed lines represent the indirect interactions. The arrows represent the interaction directions. Click here for file

Additional File 5 Top impacted pathway centralized with the cell cycle regulators by the EGCG-induced genes in the NNK-induced mouse lung tumors. Shadowed shapes represent the EGCG targeted genes. Dashed lines represent the indirect interactions. The arrows represent the interaction directions. Click here for file

Additional File 6 Top impacted pathway centralized with the regulation of MAP kinase ERK by the EGCG-induced genes in the NNK-induced mouse lung tumors. Shadowed shapes represent the EGCG targeted genes. Dashed lines represent the indirect interactions. The arrows represent the interaction directions. Click here for file

Additional File 7 The impacted pathway by the EGCG-induced miRNA targets in the NNK-induced mouse lung tumors is centralized with IGFBP5 signaling involving in the regulations of AKT and MAP kinases. Shadowed shapes represent the EGCG targeted genes. Dashed lines represent the indirect interactions. The arrows represent the interaction directions. Click here for file

## Declaration

Publication of this research was supported by the University of Saint Joseph (institutional faculty development fund awarded to HZ).

This article has been published as part of *BMC Genomics *Volume 15 Supplement 11, 2014: Selected articles from the 2014 International Conference on Advances in Big Data Analytics. The full contents of the supplement are available online at http://www.biomedcentral.com/bmcgenomics/supplements/15/S11.
