# Supplementary figures and images for: Gene regulation mediated by microRNAs in response to green tea polyphenol EGCG in mouse lung cancer
Source: BMC Genomics. 2014 Dec 16;15(Suppl 11):S3. doi: 10.1186/1471-2164-15-S11-S3 (PMC4304179; doi:10.1186/1471-2164-15-S11-S3)

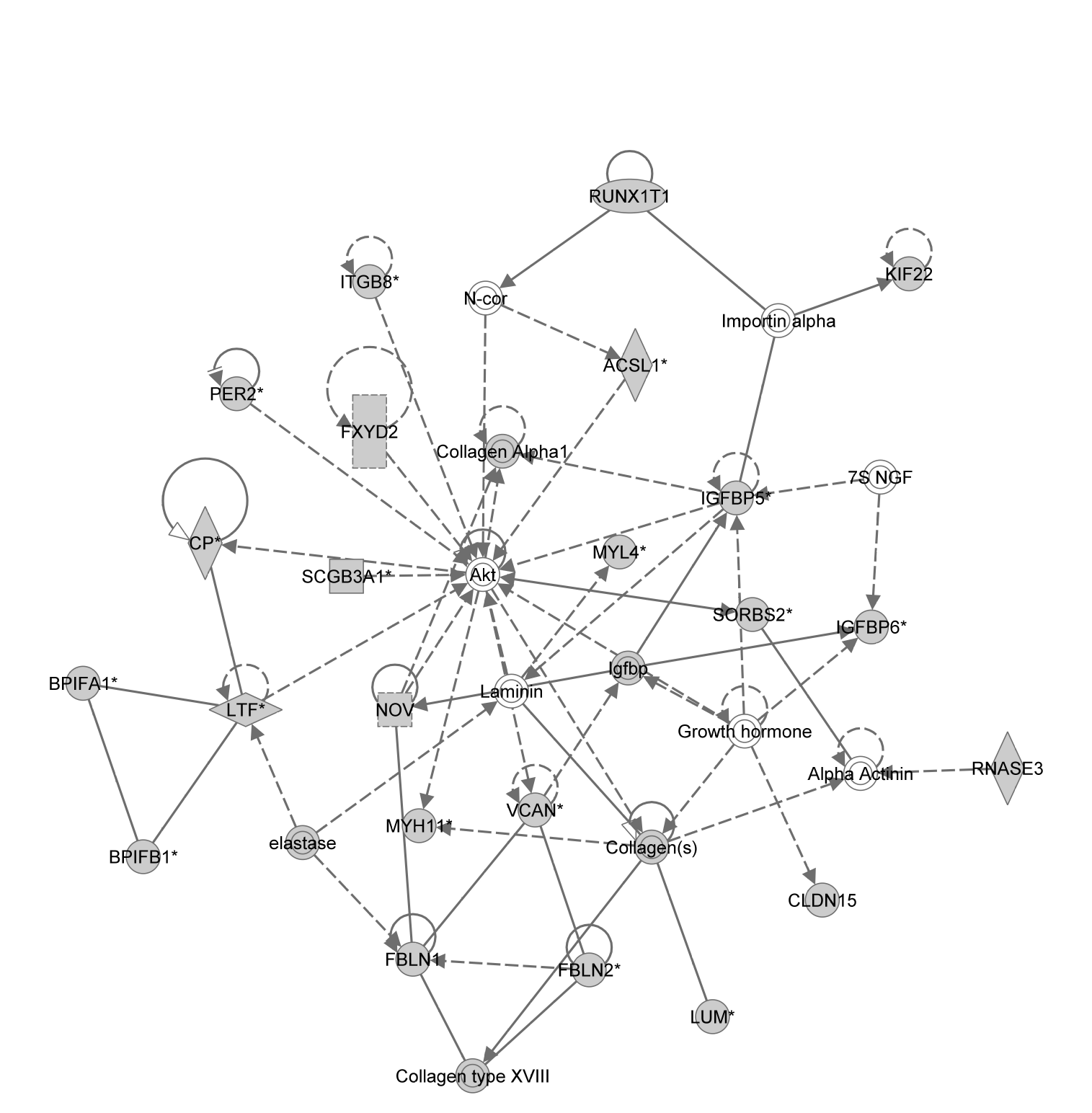

Supplement: Additional File 3 — Top impacted pathway centralized with the regulation of AKT by the EGCG-induced genes in the NNK-induced mouse lung tumors. Shadowed shapes represent the EGCG targeted genes. Dashed lines represent the indirect interactions. The arrows represent the interaction directions. [file 1471-2164-15-S11-S3-S3.tif]

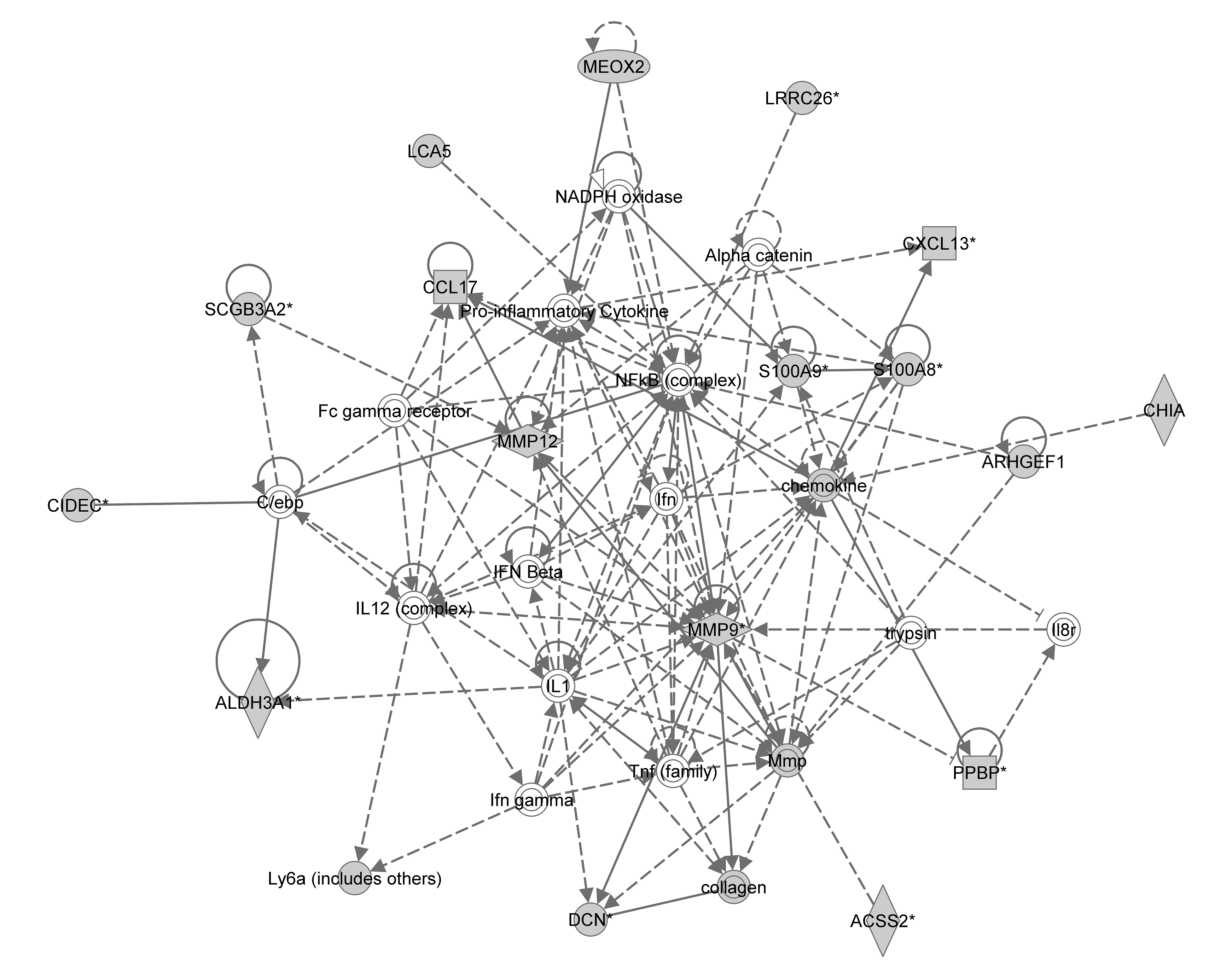

Supplement: Additional File 4 — Top impacted pathway centralized with the regulation of NF-κB and cytokines/chemokines by the EGCG-induced genes in the NNK-induced mouse lung tumors. Shadowed shapes represent the EGCG targeted genes. Dashed lines represent the indirect interactions. The arrows represent the interaction directions. [file 1471-2164-15-S11-S3-S4.tif]

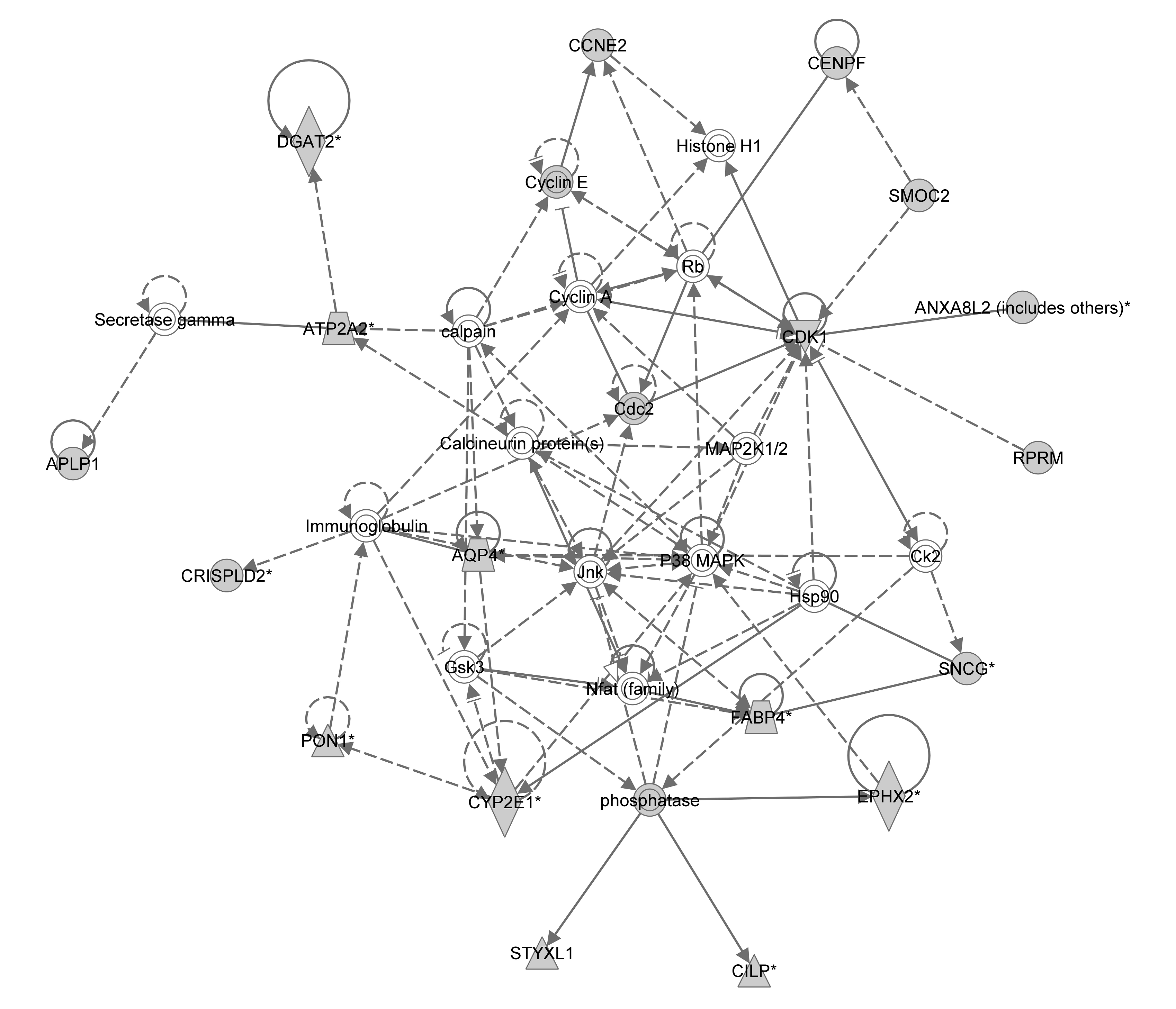

Supplement: Additional File 5 — Top impacted pathway centralized with the cell cycle regulators by the EGCG-induced genes in the NNK-induced mouse lung tumors. Shadowed shapes represent the EGCG targeted genes. Dashed lines represent the indirect interactions. The arrows represent the interaction directions. [file 1471-2164-15-S11-S3-S5.tif]

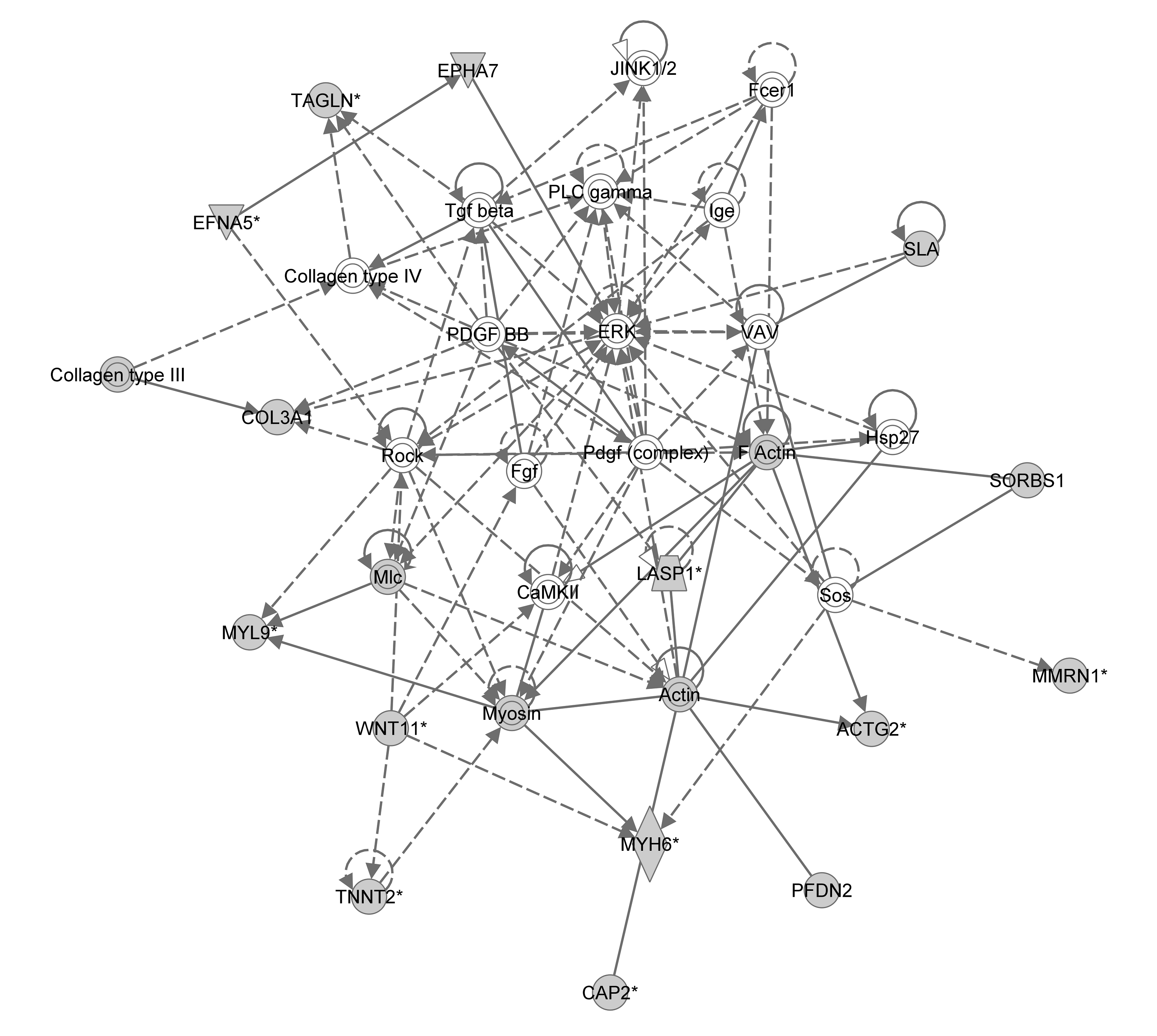

Supplement: Additional File 6 — Top impacted pathway centralized with the regulation of MAP kinase ERK by the EGCG-induced genes in the NNK-induced mouse lung tumors. Shadowed shapes represent the EGCG targeted genes. Dashed lines represent the indirect interactions. The arrows represent the interaction directions. [file 1471-2164-15-S11-S3-S6.tif]

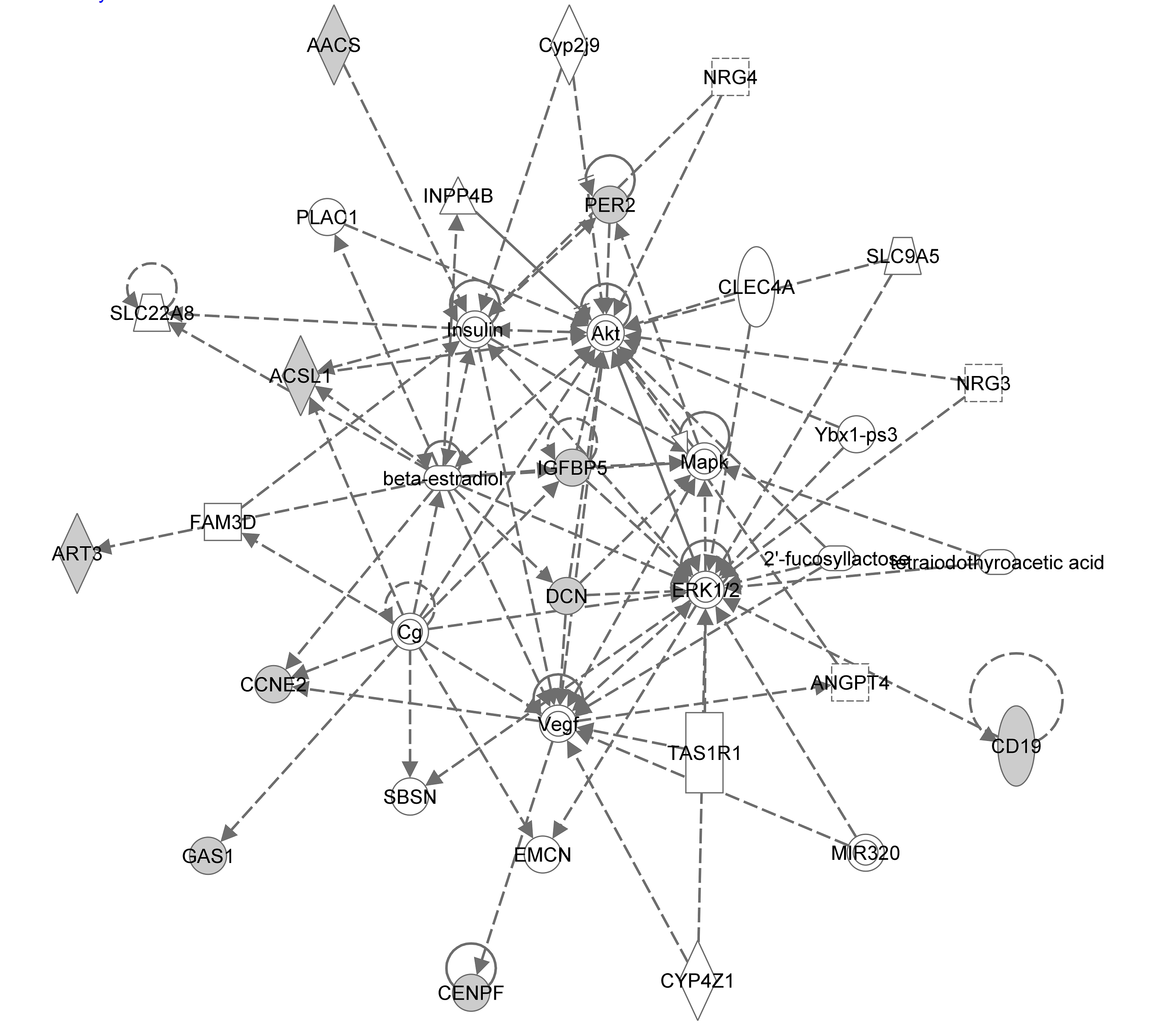

Supplement: Additional File 7 — The impacted pathway by the EGCG-induced miRNA targets in the NNK-induced mouse lung tumors is centralized with IGFBP5 signaling involving in the regulations of AKT and MAP kinases. Shadowed shapes represent the EGCG targeted genes. Dashed lines represent the indirect interactions. The arrows represent the interaction directions. [file 1471-2164-15-S11-S3-S7.tif]
